# 1,25-D3 attenuates cerebral ischemia injury by regulating mitochondrial metabolism *via* the AMPK/AKT/GSK3β pathway

**DOI:** 10.3389/fnagi.2022.1015453

**Published:** 2022-10-17

**Authors:** Yutian Li, Xiangling Li, Shuangli Xu, Yingzhe Zhao, Meng Pang, Xiaojun Zhang, Xuejian Wang, Yanqiang Wang

**Affiliations:** ^1^School of Pharmacy, Weifang Medical University, Weifang, China; ^2^Department of Internal Medicine, The Affiliated Hospital of Weifang Medical University, Weifang, China; ^3^Emergency Department, The Affiliated Hospital of Weifang Medical University, Weifang, China; ^4^Department of Neurology II, The Affiliated Hospital of Weifang Medical University, Weifang, China

**Keywords:** 1, 25-D3, cerebral ischemia-reperfusion, MCAO/R, P53, caspase-3, vitamin D receptor, mitochondrial metabolism, cytochrome C

## Abstract

The brain injury caused by cerebral ischemia-reperfusion is related to mitochondrial damage. Maintaining the normal function of mitochondria, promoting angiogenesis, protecting neuronal cells, and resisting oxidative stress are the keys to functional recovery after acute ischemic stroke. In this study, we established a middle cerebral artery occlusion (MCAO) model and investigated the effects of 1α,25-dihydroxyvitamin D3 (VitD or 1,25-D3) on mitochondrial function *via* the adenosine 5'-monophosphate-activated protein kinase (AMPK)/protein kinase B (AKT)/glycogen synthase kinase-3β (GSK-3β) signaling pathway in rats with cerebral ischemia-reperfusion injury. The neurological function and infarct size were measured in each group. Hematoxylin-eosin, neuronal nucleus, and Nissl staining procedures were conducted to observe the morphology and number of the cerebral cortical neurons. Western blotting was then used to analyze p-AMPK, vitamin D receptor (VDR), p-GSK-3β, p-AKT, P53, cytochrome C (CytC), TGF-β, and vascular endothelial growth factor (VEGF) in mitochondria. Immunofluorescence staining was used to observe the expression of CytC and caspase-3. Succinate dehydrogenase, ATPase, reactive oxygen species, and malondialdehyde were detected by kits. RT-qPCR was used to analyze TGF-β, VEGF, P53, and CytC mRNA. The results revealed that the cerebral infarct volume, neurological function score, apoptotic protein P53, CytC, caspase-3, reactive oxygen species, and malondialdehyde were significantly increased in MCAO rats. 1,25-D3 reduced the infarct size and neurological function score, activated VDR, upregulated TGF-β, p-AMPK, p-AKT, p-GSK-3β, VEGF, ATP, and succinate dehydrogenase, and downregulated P53, CytC, caspase-3, reactive oxygen species, and malondialdehyde. As an antagonist of VDRs, pyridoxal-5-phosphate could partially block the neuroprotective effect of 1,25-D3. In conclusion, 1,25-D3 activated AMPK/AKT/GSK-3β signaling and VDRs, inhibited P53, CytC, and caspase-3, increased TGF-β and VEGF, regulated mitochondrial metabolism, reduced neuronal apoptosis, promoted vascular growth, and exerted neuroprotective effects. These findings suggest that this signaling pathway may be an effective target for the treatment of ischemic stroke.

## Introduction

Stroke is a serious neurological disease. There are two major types of stroke: ischemic and hemorrhagic stroke. Ischemic stroke is more common and accounts for 80% of stroke cases (Moskowitz et al., 2010). To date, early restoration of blood flow is the best treatment for ischemic stroke in clinical practice. However, restoration can exacerbate tissue damage accompanied by comparatively serious pathological reactions, which is called cerebral ischemia-reperfusion injury (Segura et al., 2008). The pathogenesis of ischemic stroke is complex and has been associated with various internal and external factors, such as energy metabolism disorder, an increase in reactive oxygen species (ROS), calcium overload, apoptosis, autophagy, inflammatory response, disturbance in the production of high energy phosphate compounds, and excitatory amino acid toxicity (Pang et al., 2014). All factors interact and eventually lead to the destruction of nerve function and the formation of cerebral infarction. Previous studies have found that mitochondrial dysfunction plays a vital part in the pathological process of ischemic stroke (Wu et al., 2017). Abnormal mitochondrial function can lead to neurological dysfunction, enhance the expression of pro-apoptosis-related factors, and activate the mitochondrial apoptosis pathway in ischemic stroke. Oxidative stress occurs when the level of ROS produced is higher than the rate at which they are removed by the antioxidant defense system. Oxidative damage to proteins, lipids, and DNA is induced by excessive ROS accumulation after stroke, and leads to apoptosis (Olmez and Ozyurt, 2012). Vitamin D has been reported to improve neuronal survival by modulating calcium content and reducing free radical production. Vitamin D has also been shown to reduce markers of oxidative stress in both *in vitro* and *in vivo* studies (Cui et al., 2019). When mitochondrial dysfunction occurs after ischemic stroke, P53 accumulates in the cytoplasmic membrane and activates the multidomain pro-apoptotic protein Bax (Chen et al., 2015); this allows Bax to interact with the voltage-dependent ion channels on mitochondria, thereby mediating the release of cytochrome C (CytC). P53 can also act directly on the mitochondrial membrane to induce the release of CytC into the cytoplasm, where it binds to Apaf-1 and then activates the apoptosis of caspase-3/9, which initiates the caspase cascade (Zhang et al., 2013) and finally induces apoptosis.

Adenosine 5'-monophosphate-activated protein kinase (AMPK) signaling affects a variety of physiological and pathological activities, such as cell metabolism, energy homeostasis, and apoptosis (Qi and Young, 2015). Importantly, activation of AMPK has been shown to confer a robust protective effect against reperfusion injury (Kubli and Gustafsson, 2014). In addition, protein kinase B(AKT)signaling serves as a key downstream target of AMPK (Kubli and Gustafsson, 2014; Daskalopoulos et al., 2016). AKT and its downstream targets glycogen synthase kinase-3β (GSK-3β) and hypoxia-inducible factor-1α have also been found to play key roles in ischemia-reperfusion injury (Kalakech et al., 2013; Huang et al., 2016; Li et al., 2019). GSK-3β/P53 plays an important role in mitochondrial apoptosis (Li et al., 2019). Moreover, 1,25-D3 is widely expressed in human organs and tissues and acts as a steroid throughout the body. 1,25-D3 binds to the vitamin D receptor (VDR) and produces a wide range of biological activities, including inhibition of proliferation, effects on angiogenesis, and regulation of immune and endocrine activity (Brozyna et al., 2014; Mori et al., 2020). 1,25-D3 has been found to prevent ischemic injury and improve stroke 3 days after middle cerebral artery occlusion (MCAO) (Zhang et al., 2022).

Observational research has indicated that patients with low serum 1,25-D3 levels have poor infarct volume and functional prognosis after stroke (Daubail et al., 2013), which suggests that 1,25-D3 may play a protective role in cerebral ischemia; however, the role of 1,25-D3 in mitochondrial metabolism, cell apoptosis, and the recovery of neurological function has not yet been investigated. The objective of this study was to explore the protective mechanism of 1,25-D3 against brain injury and to determine the role of AMPK/AKT/GSK-3β signaling in this process, thus allowing us to determine the therapeutic potential of 1,25-D3 in stroke.

## Materials and methods

### Animals

This study was performed using adult male Sprague-Dawley rats weighing 280–330 g. Rats were provided by the Experimental Animal Center of Weifang Medical University, license SCXK(LU)20190003. Animals were kept in pathogen-free conditions and housed in a standard environment (22 ± 2°C) under a 12-h light/dark cycle with a humidity of 50–60% throughout the experiment, and had *ad libitum* access to food and water. All experiments were approved by the Ethics Committee for the Use of Experimental Animals at Weifang Medical University (2018-037).

### Chemicals and reagents

The compounds 1,25-D3, VDR antagonist pyridoxal-5-phosphate (P5P), and 2,3,5-triphenyltetrazolium chloride (TTC) were purchased from Sigma-Aldrich (St. Louis., MO, USA). The primary antibodies anti-VDR antibody (14526-1-AP) and anti-β-actin antibody (20536-1-AP) were purchased from Proteintech (Rosemont, IL, USA). Anti-VEGF antibody (ab1316) and anti-CytC antibody (ab133504) were purchased from Abcam (Cambridge, MA, USA). The primary antibodies anti-COXIV(4850), anti-AMPK (5832), anti-p-AMPK (2535), anti-AKT (4685), anti-p-AKT (4060), anti-GSK-3β (9315), anti-p-GSK-3β (9336), anti-TGF-β (3711S), and anti-P53 (2524) were purchased from Cell Signaling Technology (Danvers, MA, USA).

Secondary antibodies HRP-conjugated AffiniPure Goat Anti-Rabbit (SA00001-2) and HRP-conjugated AffiniPure Goat Anti-Mouse (SA00001-1) were purchased from Proteintech. MCAO surgical nylon monofilaments were 0.38–0.40 mm MSRC40B200PK50 (Shen Zhen RWD Life Science Co., Ltd.).

### Establishment of the MCAO rat model and grouping

Eighty male Sprague-Dawley rats were randomly divided into four groups with 20 rats in each group: Sham, MCAO, MCAO + 1,25-D3, and MCAO+1,25-D3+P5P. Anesthesia was performed by intraperitoneal injection of 1% pentobarbital sodium (50 mg/kg). The cortex was dissected to separate the arteria carotis communis, and external and internal carotid arteries, and surgical nylon monofilament was inserted into the right internal carotid artery at the bifurcation until slight resistance was encountered. After 90 min, the monofilament was pulled out, and then the nylon monofilament was removed and reperfused for 6, 12, 24 h, and 3 days. The Sham group did not have the nylon monofilament inserted. 1,25-D3 and 1,25-D3 +P5P groups were intravenously injected with 1,25-D3 and 1,25-D3 +P5P once a day for 7 consecutive days before MCAO induction. 1,25-D3 was dissolved in 2% DMSO and administered intravenously (5 μg/kg) 30 min before surgery, and P5P was dissolved in 2% DMSO and administered intravenously (0.5 μg/kg) 60 min before surgery. 1,25-D3 and P5P were given once a day for the next 3 consecutive days. After the last dose, rats were killed and brain tissue was removed for subsequent studies.

### Assessment of cerebral infarct size

The thread plugs were removed, five animals in each group were sacrificed 3 days after reperfusion, and the brains were removed and frozen at −20°C for 30 min. The brains were sectioned into 2-mm sections and then stained with TTC. The samples were incubated for 30 min in 2% TTC solution at 37°C, washed in PBS, and then the liquid was discarded. Then the sections were lined up sequentially, photographed, and analyzed using ImageJ software (National Institutes of Health, Bethesda, USA). The percentage of relative infarction volume was calculated by the following formula: Infarct size = (volume of the contralateral hemisphere – volume of the ipsilateral hemisphere non-infarct)/volume of the contralateral hemisphere × 100%.

### Neurological functional assessment

The modified Neurological Severity Scale (mNSS) score was used to assess the neurological function of the rats at 6, 12, 24 h, and 3 days after reperfusion. A total of 18 items were included in this scale, based on the tests of reflexes, sensory perception, motor planning, and beam balance (normal score = 0; maximum deficit score = 18). All surgical procedures were performed after the rats had acclimated to the testing environment.

### Hematoxylin-eosin staining and Nissl's staining

Rats were anesthetized and sacrificed. After anesthesia, pre-cooled 0.9% NaCl was perfused transapically, followed by 4% paraformaldehyde at pH 7.4. The skull was dissected, brain tissues were removed and soaked in 4% paraformaldehyde overnight, and then embedded in paraffin and cut into 5-μm-thick sections using a microtome. Sections were baked at 55°C for about 30 min, dewaxed with xylene, dehydrated with gradient alcohol, washed with running water for 1 min, and stained with HE and toluidine blue for 12 min at room temperature (RT). Then, the sections were rinsed with running water, differentiated with 1% alcohol hydrochloride, and rinsed again. Next, the sections were restored to blue in saturated lithium carbonate solution for 25 s and stained with HE solution for 2 min. Finally, the stained sections were dehydrated with alcohol, transparented with xylene, and sealed with neutral balata.

### NeuN immunohistochemical staining

After dewaxing and dehydration, sections were incubated with 3% hydrogen peroxide at RT for 10 min, followed by washing with PBS for three times. Then, the sections were incubated with anti-NeuN (ab177487, 1/1000, Abcam, Cambridge, UK) at 37°C for 1 h, washed with PBS again for three times, added horseradish peroxidase, and kept at 37°C for 40 min. Then, 3, 3′-diaminobenzidine (DAB) and hematoxylin staining was done and sealed.

### Western blot analysis

In brain tissue, the expression levels of VDR, vascular endothelial growth factor (VEGF), TGF-β, P53, and CytC were measured using Western blotting. The brain tissues were lysed in precooled RIPA lysis buffer (pyrolysis liquid) for 30 min (Beyotime). A mitochondrial extraction kit (Beyotime) was used for total protein extraction from mitochondria. The supernatant was aspirated after centrifugation. The concentrations of all the proteins were determined using the BCA protein assay kit (Beyotime). We then performed protein denaturation at 100°C with the loading buffer. According to the molecular weight of different proteins, the appropriate separation gel was selected for electrophoresis. Next, the protein was transferred to a nitrocellulose membrane, which was incubated in 5% skimmed milk for 1 h at room temperature. Each membrane was then washed three times for 10 min using Tris-HCL buffer salt solution and Tween20 (TBST), and incubated in diluted primary antibodies overnight at 4°C. The proportion of primary antibodies was as follows: VDR (1:2,000), AMPK (1:1,000), p-AMPK (1:1,000), AKT (1:1,000), p-AKT (1:1,000), GSK-3β (1:1,000), p-GSK-3β (1:1,000), P53 (1:1,000), CytC (1:5,000), VEGF (1:1,000), and TGF-β (1:1,000). After washing the membrane three times for 10 min with Tris-HCL buffer salt solution and Tween 20, the membranes were incubated with secondary antibodies (1:5,000) for 2 h at room temperature. The enhanced chemiluminescence method was used to display the bands, and ImageJ software was used to analyze the gray value of the bands. The gray value of the target protein band/β-actin gray value was used to determine the expression of the target protein band.

### Immunofluorescence staining

Wax chunks from the previous step of the experiment were taken and cut into slices. The slices were first incubated with the 10% bovine serum albumin for 2 h and then co-incubated with the antibody at 4°C overnight. After washing in phosphate-buffered saline (PBS), the water was wiped off, and the tissue sections were incubated with the secondary antibodies for 1 h in the dark. The stained sections were observed with a fluorescence microscope and analyzed using ImageJ software.

### Enzyme activity assay

The same mass of tissue was accurately weighed for each group, and nine times more normal saline was added at a ratio of 1:9. The homogenate was mechanically homogenized on ice to prepare 10% homogenate and centrifuged for 10 min, and 0.2 ml of supernatant was taken and 0.8 ml of normal saline was diluted in 2% homogenate to be tested. Next, mitochondrial enzyme activities were assayed according to the instructions of ROS Assay Kit (Affandi, Shanghai, China) and malondialdehyde (MDA), ATP, succinate dehydrogenase (SDH) Assay Kit (JianCheng, Nanjing, China), respectively.

### Quantitative real-time PCR

The brain cortex of the ischemic side of each rat was separated and placed in an enzyme-free tube that had been prepared in advance. The RNA extraction Kit (Beyotime, Shanghai, China) was used to extract total RNA, wherein 20 μl of reverse transcription system was mixed with 1 μg of RNA template. The RNA samples were reverse-transcribed to cDNA in a machine at 50°C for 15 min and 85°C for 5 min. After the reaction, the samples were briefly centrifuged and cooled on ice. PCR was performed in a PCR machine. About 20 μl of amplification solution was mixed with 1 μl of cDNA and 10 μl of fluorescent dye. Samples then underwent the following protocol: denaturation at 95°C for 10 min and 40 cycles at 95°C for 15 s and 60°C for 60 s. The relative mRNA level of the gene was measured *via* 2^−ΔΔCT^ method. GAPDH was used as the endogenous reference gene. HiFiScript cDNA Synthesis Kit and UltraSYBR Mixture were purchased from CWBIO (Taizhou, China). The primer sequences are listed in Table 1.

**Table 1:** PCR primers.

**Type**	**Primer**
VDR	Forward 5′-CCACCGGCAGAAACGTGTAT-3′,
VDR	Reverse 5′-TGCCTTGTGAGAGGCTCTAGGA-3′,
VEGF	Forward 5′-CCGTCCTGTGTGCCCCTAATG-3′,
VEGF	Reverse 5′-CGCATGATCTGCATAGTGACGTTG-3′,
TGF-β	Forward 5′-TGAGTGGCTGTCTTTTGACG-3′,
TGF-β	Reverse 5′-GGTTCATGTCATGGATGGTG-3′,
P53	Forward 5′-TCCCCCTTGCCGTCCCAA-3′,
P53	Reverse 5′-CGTGCAAGTCACAGACTT-3′

### Statistical analysis

GraphPad Prism 8 (Graph Pad Software Inc, San Diego, CA, USA) was used to generate graphs and perform the statistical analysis. All experimental data obtained are expressed as the mean ± SEM. A one-way ANOVA was used for comparisons between multiple groups, and Tukey's *post-hoc* test was used for further pairwise comparisons. Differences were considered statistically significant at *P* < 0.05.

## Results

### Treatment with 1,25-D3 significantly diminished the volume of early brain injury infarct regions after MCAO/R

**Figure 1:**
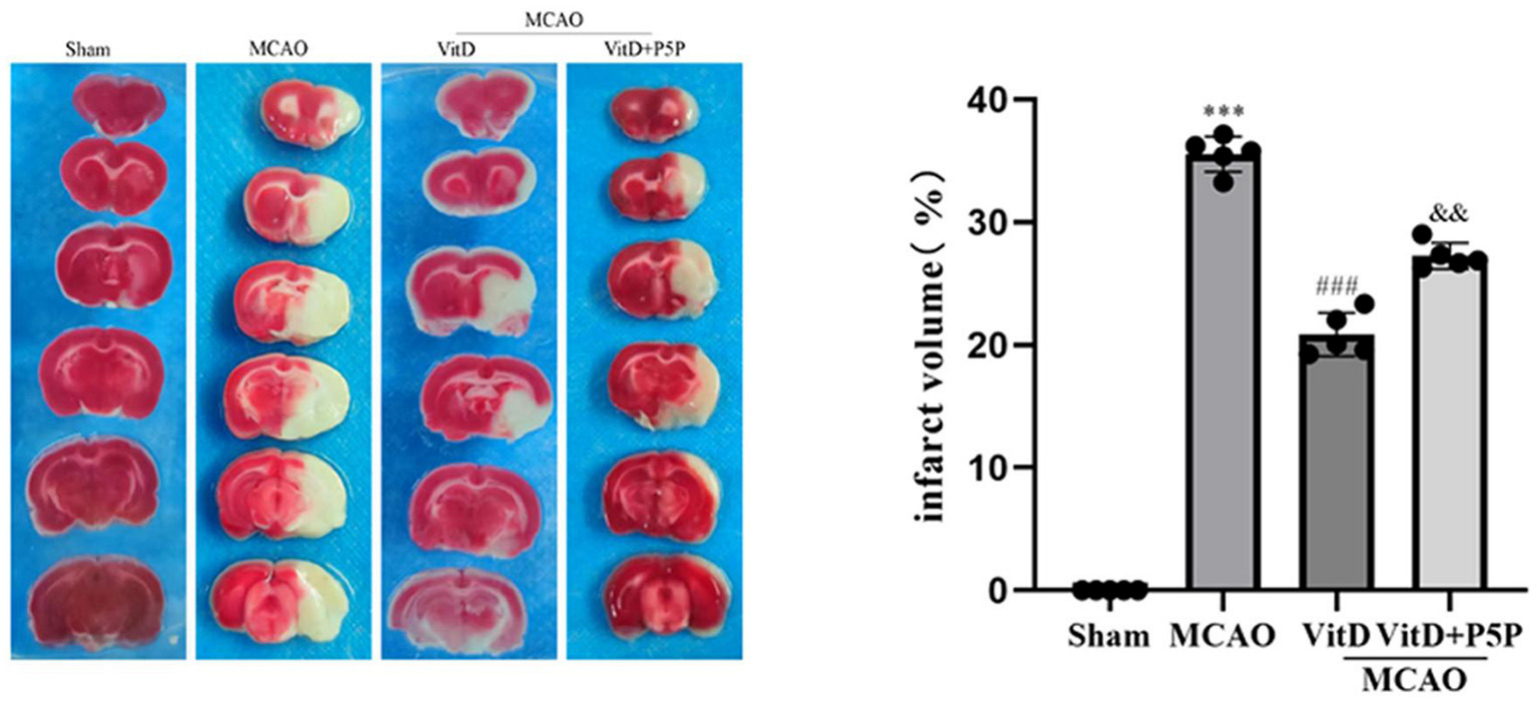
1,25-D3 reduced the volume of infarct regions in rats 3 days after stroke. All values are the mean ± SEM, ***P < 0.001 (vs. Sham), ^*###*^*P* < 0.001 (vs. MCAO), and ^&&^*P* < 0.01, (vs. VitD), *n* = 5. P5P, antagonist of VDR; Vit D, (1,25-D3).

To evaluate the protective effect of 1,25-D3 against cerebral ischemia-reperfusion, we analyzed cerebral infarct volume 3 days after MCAO. There was a significant difference between groups (F = 2512, *P* < 0.0001). The infarct volume of the MCAO group was significantly larger than that of the Sham group (*P* < 0.001). The infarct volume of the 1,25-D3 group was smaller than that of the MCAO group (*P* < 0.001). However, the infarct volume of the P5P group was larger than that of the 1,25-D3 group (*P* < 0.01) (Figure 1). These results demonstrate that administration of 1,25-D3 significantly reduced the infarct volume and played a protective role in neurological function.

### 1,25-D3 ameliorated neurobehavioral function after MCAO/R

**Figure 2:**
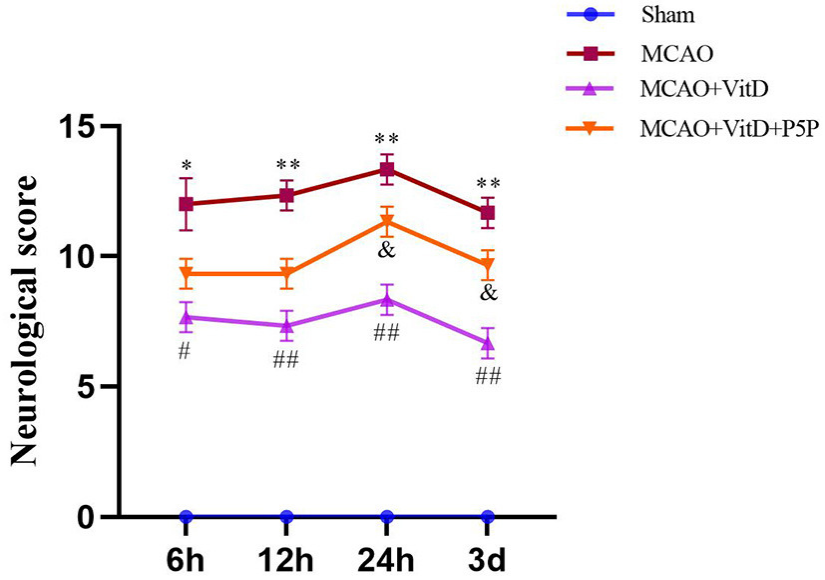
Treatment with 1,25-D3 (Vit D) improved the nerve injury induced by MCAO. The modified Neurological Severity Scale scores were assessed at 6, 12, 24 h, and 3 days after reperfusion. All values are shown as the mean ± SEM, *P < 0.05, **P < 0.01 (vs. Sham), ^#^*P* < 0.05, ^*##*^*P* < 0.01 (vs. MCAO), ^&^*P* < 0.05 (vs. VitD), *n* = 5. P5P, antagonist of VDR; Vit D, (1,25-D3).

To determine the effect of 1,25-D3 on brain injury, the modified Neurological Severity Scale score of rats was evaluated at 6, 12, 24 h, and 3 days after reperfusion. There was a significant difference between groups (F = 2.538, *P* < 0.05). The neurological function of MCAO rats was worse than that of the Sham group (*P* < 0.01). Compared with the MCAO group, treatment with 1,25-D3 significantly improved the neurological function at all time points after reperfusion and began to decline 3 days after surgery (*P* < 0.01). However, the protective effect of P5P on neural function was slightly worse than that of 1,25-D3 (*P* < 0.05; Figure 2). In conclusion, 1,25-D3 ameliorated neurological deficits.

### Effects of 1,25-D3 on the morphology of cortical neurons in rats with MCAO/R

**Figure 3:**
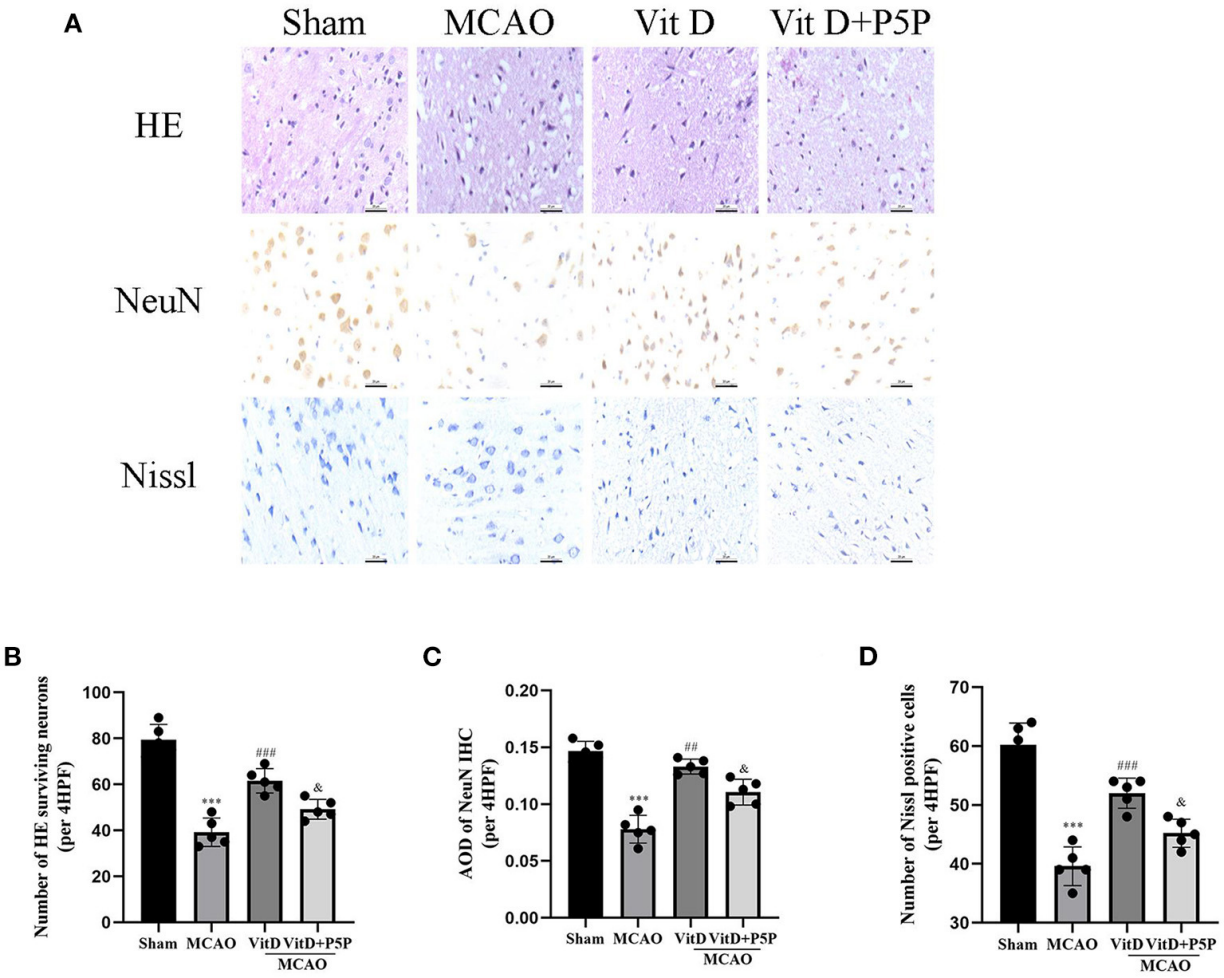
HE, NeuN, and Nissl staining of cortical neurons of each group. **(A)** HE, NeuN, and Nissl staining procedures were conducted to observe the morphology and number of the cerebral cortical neurons (400×). **(B–D)** Quantification of the HE, NeuN, and Nissl staining. All values are the mean ± SEM. ***P < 0.001 (vs. Sham), ^*##*^*P* < 0.01, ^*###*^*P* < 0.001 (vs. MCAO), ^&^*P* < 0.05 (vs. VitD), *n* = 5. P5P, antagonist of VDR; Vit D, (1,25-D3).

There were significant differences in the number of cerebral cortical neurons between groups (HE, F = 46.16, *P* < 0.0001). Compared with the Sham group, which showed no cerebral cortex edema with normal neurons and visible Nissl bodies, the MCAO group showed edema of cerebral cortex neurons, necrosis of a large number of neurons, and irregularly arranged Nissl bodies (HE, *P* < 0.001; NeuN, *P* < 0.001; Nissl, *P* < 0.001). In the 1,25-D3 group, there was partial edema of cerebral cortex neurons, a small amount of neuronal necrosis, and an increase in visible Nissl bodies (HE, *P* < 0.001; NeuN, *P* < 0.01; Nissl, *P* < 0.001). The P5P group was inferior to the 1,25-D3 group (HE, *P* < 0.05; NeuN, *P* < 0.05; Nissl, *P* < 0.05), with partial edema, moderate amount of neuronal necrosis, and some irregular patches of Nissl bodies (NeuN, F = 45.9, *P* < 0.0001). Compared with the Sham group, the 1,25-D3 group had a lower number of Nissl-positive and NeuN-positive neurons (Nissl, F = 43.05, *P* < 0.0001). These results indicate that 1,25-D3 can reduce edema, reduce neuronal necrosis, and increase Nissl bodies, which is helpful for partial recovery of the infarct site and for slowing down the process of necrosis (Figure 3).

### The AMPK/AKT/GSK-3β pathway regulated mitochondrial apoptosis-related protein expression

**Figure 4:**
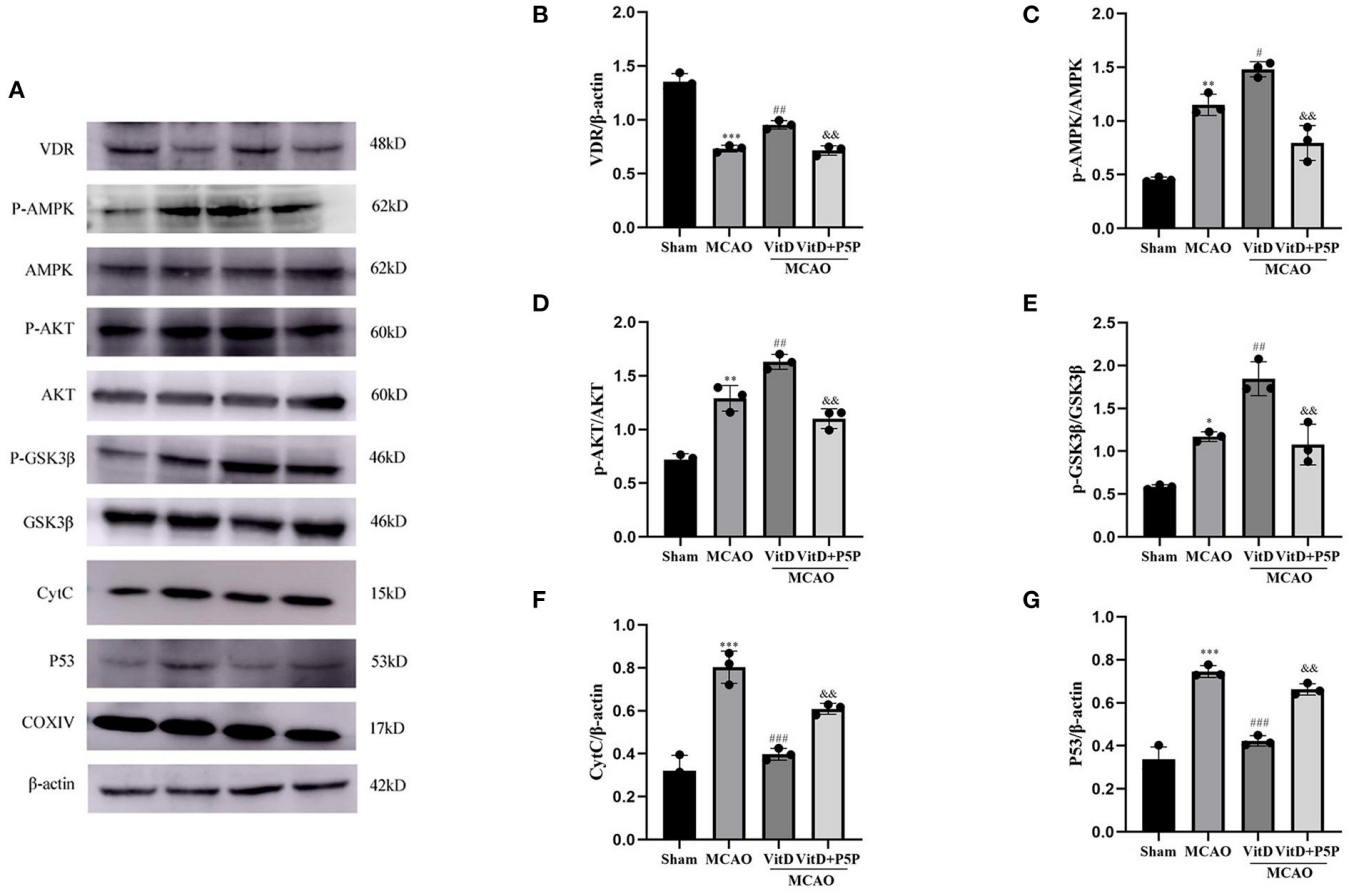
**(A)** Images of Western blot for VDR, the AMPK/AKT/GSK-3β pathway, P53, and CytC. **(B–G)** The expression levels of CytC, VDR, p-AMPK, p-AKT, p-GSK-3β, and P53 in the brain tissues of different groups were analyzed using ImageJ. All values are shown as the mean ± SEM. *P < 0.05, **P < 0.01, ***P < 0.001 (vs. Sham), ^#^*P* < 0.05, ^*##*^*P* < 0.01, ^*###*^*P* < 0.001 (vs. MCAO), ^&&^*P* < 0.01 (vs. VitD), *n* = 5. MCAO, middle cerebral artery occlusion; P5P, antagonist of VDR; Vit D, (1,25-D3).

To determine the effect of 1,25-D3 on mitochondrial apoptosis *via* the AMPK/AKT/GSK-3β signaling pathway, we analyzed the protein changes in the pathway. There were significant between-group differences in proteins (p-AMPK, F = 53.47, *P* < 0.001; p-AKT, F = 56.16, *P* < 0.0001; p-GSK-3β, F = 32.59, *P* < 0.0001). Compared with the Sham group, the expression levels of p-AMPK, p-AKT, and p-GSK-3β were higher in the MCAO group (*P* < 0.01, *P* < 0.01, *P* < 0.05, respectively), and these levels were even higher in the 1,25-D3 group (*P* < 0.05, *P* < 0.01, *P* < 0.01, respectively). P5P in turn inhibited the effect of 1,25-D3, but the expression levels of p-AMPK and p-AKT (*P* < 0.01, *P* < 0.01, *P* < 0.01) were higher than those of the Sham group (VDR, F = 105.9, *P* < 0.0001; P53, F = 84.02, *P* < 0.0001; CytC, F = 47.02, *P* < 0.0001). Compared with the Sham group, the expression level of VDR was decreased (*P* < 0.001), but the levels of P53 and CytC were increased in the MCAO group (*P* < 0.001, *P* < 0.001). The administration of 1,25-D3 activated VDR, and inhibited the expression of mitochondrial apoptosis proteins P53 and CytC (*P* < 0.001, *P* < 0.001). P5P partially reversed the protective effect of 1,25-D3 on mitochondrial function, the expression levels of P53 and CytC were higher than those of the 1,25-D3 group (*P* < 0.01, *P* < 0.01, respectively), and VDR was lower than in the 1,25-D3 group (*P* < 0.01). These results indicate that 1,25-D3 can reduce the expression of mitochondrial apoptotic proteins P53 and CytC through the AMPK/AKT/GSK-3β pathway, thereby reducing neuronal cell apoptosis and alleviating brain injury (Figure 4).

### 1,25-D3 decreased the mitochondrial apoptosis of neurons in rats with MCAO/R

**Figure 5:**
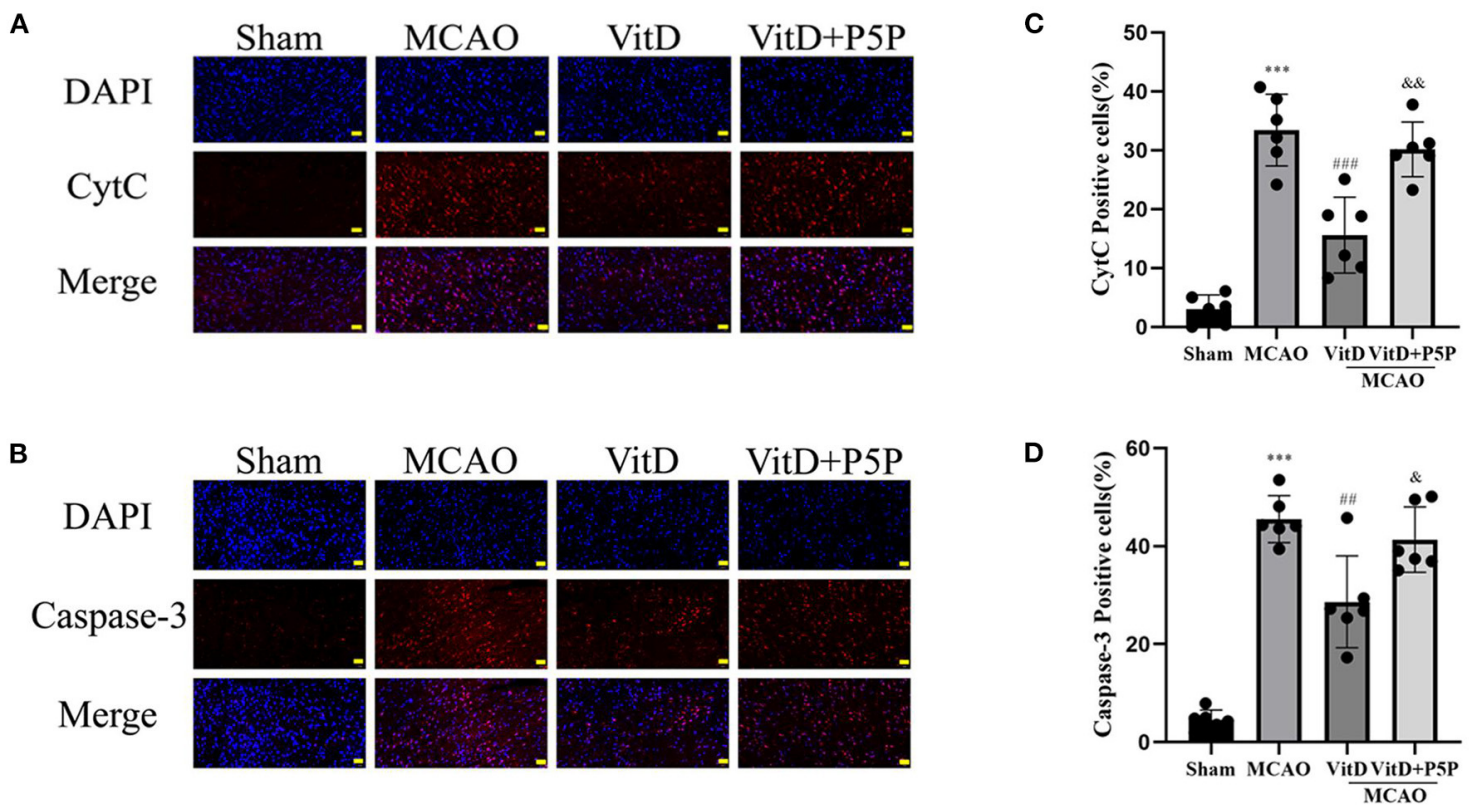
1,25-D3 (Vit D) can inhibit the expression of mitochondrial apoptotic proteins after cerebral ischemia-reperfusion injury. **(A,B)** *In situ* expression of CytC (red), caspase-3 (red) in the peri-infarct region of sham-operated, MCAO/R, DMSO, 1,25-D3, and P5P groups. DAPI (blue). **(C,D)** The percentage of positive cells was calculated. Scale bar = 50 μm. All values are shown as the mean ± SEM. ***P < 0.001 (vs. Sham), ^*##*^*P* < 0.01, ^*###*^*P* < 0.001 (vs. MCAO), ^&^*P* < 0.05, ^&&^*P* < 0.01 (vs. VitD), *n* = 5. P5P, antagonist of VDR; Vit D, (1,25-D3).

To further elucidate the mechanism underlying the protective effect of 1,25-D3, we determined the levels of apoptotic proteins caspase-3 and CytC after brain injury. There was a significant difference between groups (CytC, F = 44.57, *P* < 0.0001; caspase-3, F = 51.13, *P* < 0.0001). The immunofluorescence results demonstrated that the expression levels of caspase-3 and CytC in the MCAO group were higher than those in the Sham group (*P* < 0.001, *P* < 0.001, respectively), and the expression levels of caspase-3 and CytC were significantly decreased after the administration of 1,25-D3 (*P* < 0.01, *P* < 0.001, respectively). P5P partially inhibited the expression of caspase-3 and CytC (*P* < 0.05, *P* < 0.01, respectively), which was higher than that in the 1,25-D3 group. Therefore, this finding once again confirmed the inhibitory effect of 1,25-D3 on mitochondrial apoptotic proteins after brain injury (Figure 5).

### 1,25-D3 attenuated cerebral ischemia injury *via* regulation of mitochondrial metabolism and promoted angiogenesis

**Figure 6:**
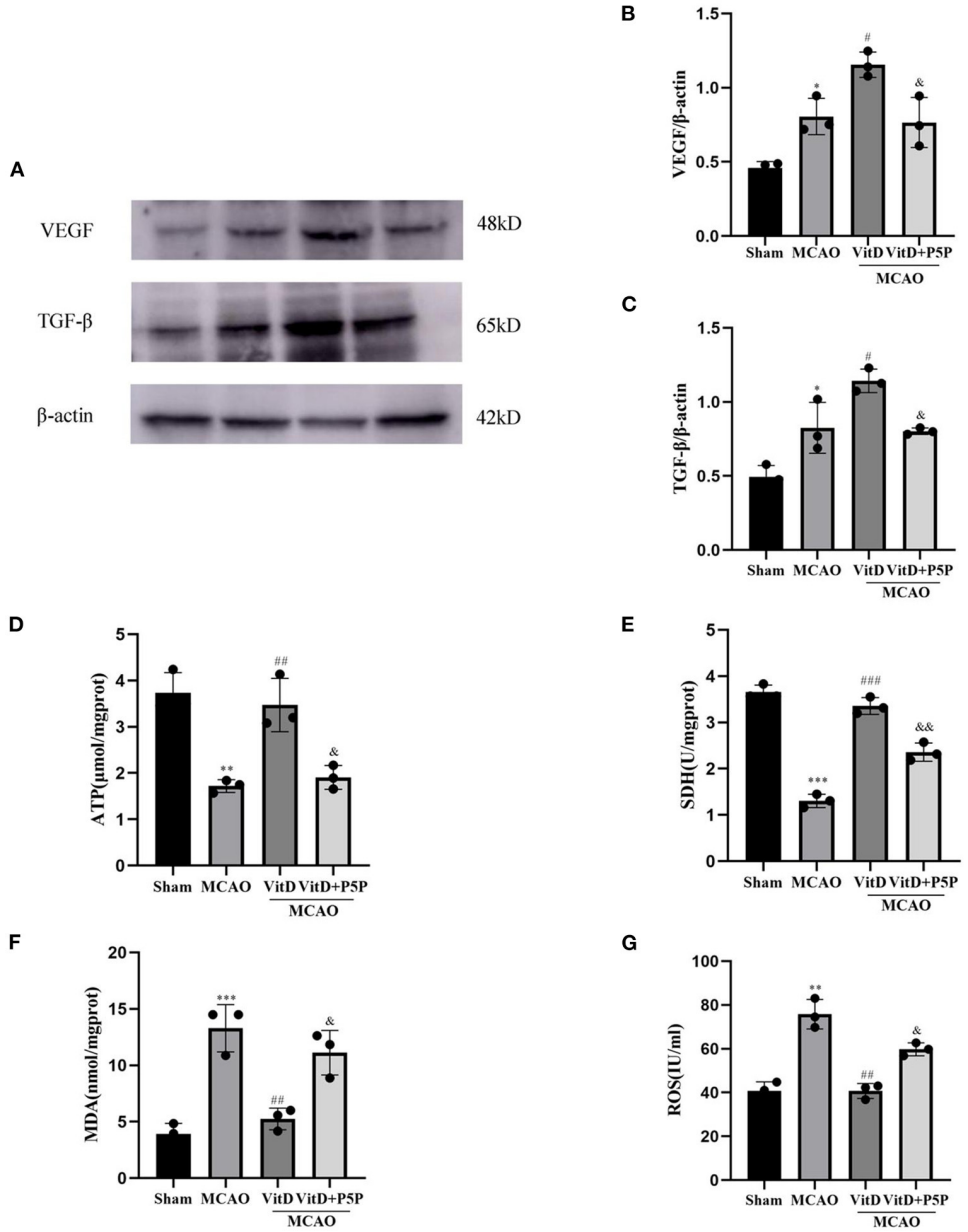
1,25-D3 regulated the activities of mitochondrial ATPase and SDH, reduced the levels of ROS and MDA, and promoted the expression of TGF-β and VEGF. **(A)** Western blot images for TGF-β and VEGF. **(B,C)** The gray value of the band was analyzed using ImageJ. **(D–G)** ATPase and SDH, MDA, and ROS expression levels in mitochondria. All values are shown as the mean ± SEM. *P < 0.05, **P < 0.01, ***P < 0.001 (vs. Sham), ^#^*P* < 0.05, ^*##*^*P* < 0.01, ^*###*^*P* < 0.001 (vs. MCAO), ^&^ *P* < 0.05, ^&&^*P* < 0.01 (vs. VitD), *n* = 5. P5P, antagonist of VDR; Vit D, (1,25-D3).

To explore the effect of 1,25-D3 on mitochondrial metabolism and angiogenesis after brain injury, the expression levels of TGF-β and VEGF were detected by Western blotting (VEGF, F = 18.50, *P* < 0.001; TGF-β, F = 20.01, *P* < 0.001). The activities of mitochondrial SDH and ATP, and the levels of ROS and MDA were measured using kits (ATP, F = 17.98, *P* < 0.01; SDH, F = 117.7, *P* < 0.0001; ROS, F = 27.66, *P* < 0.001; MDA, F = 24.30, *P* < 0.001). The expression levels of TGF-β and VEGF in the MCAO group were higher than those in the Sham group (*P* < 0.05, *P* < 0.05, respectively). 1,25-D3 further promoted the release of TGF-β and VEGF compared with MCAO (*P* < 0.05, *P* < 0.05, respectively). The P5P group inhibited the release of TGF-β and VEGF compared with 1,25-D3 (*P* < 0.05, *P* < 0.05), which resulted in a lower expression of TGF-β and VEGF than in the 1,25-D3 group. Compared with the Sham group, the levels of ROS and MDA in mitochondria were significantly higher in the MCAO group (*P* < 0.01, *P* < 0.001, respectively), while the activities of ATP and SDH were significantly lower (*P* < 0.01, *P* < 0.001, respectively). 1,25-D3 reversed this phenomenon, and reduced the levels of ROS and MDA (*P* < 0.01, *P* < 0.01, respectively), and increased the activities of ATP and SDH (*P* < 0.01, *P* < 0.001, respectively) in mitochondria. However, P5P partially inhibited the effect of 1,25-D3; the levels of ROS and MDA were higher than those in the 1,25-D3 group (*P* < 0.05, *P* < 0.05, respectively), and activities of ATP and SDH were lower than those in the 1,25-D3 group (*P* < 0.05, *P* < 0.01, respectively). In conclusion, 1,25-D3 improved ATP and SDH activities, downregulated ROS and MDA levels in mitochondria, improved mitochondrial metabolic function, and reduced mitochondrial function damage (Figure 6).

### The expression of VDR, VEGF, TGF-β, and P53 mRNA was affected by 1,25-D3

**Figure 7:**
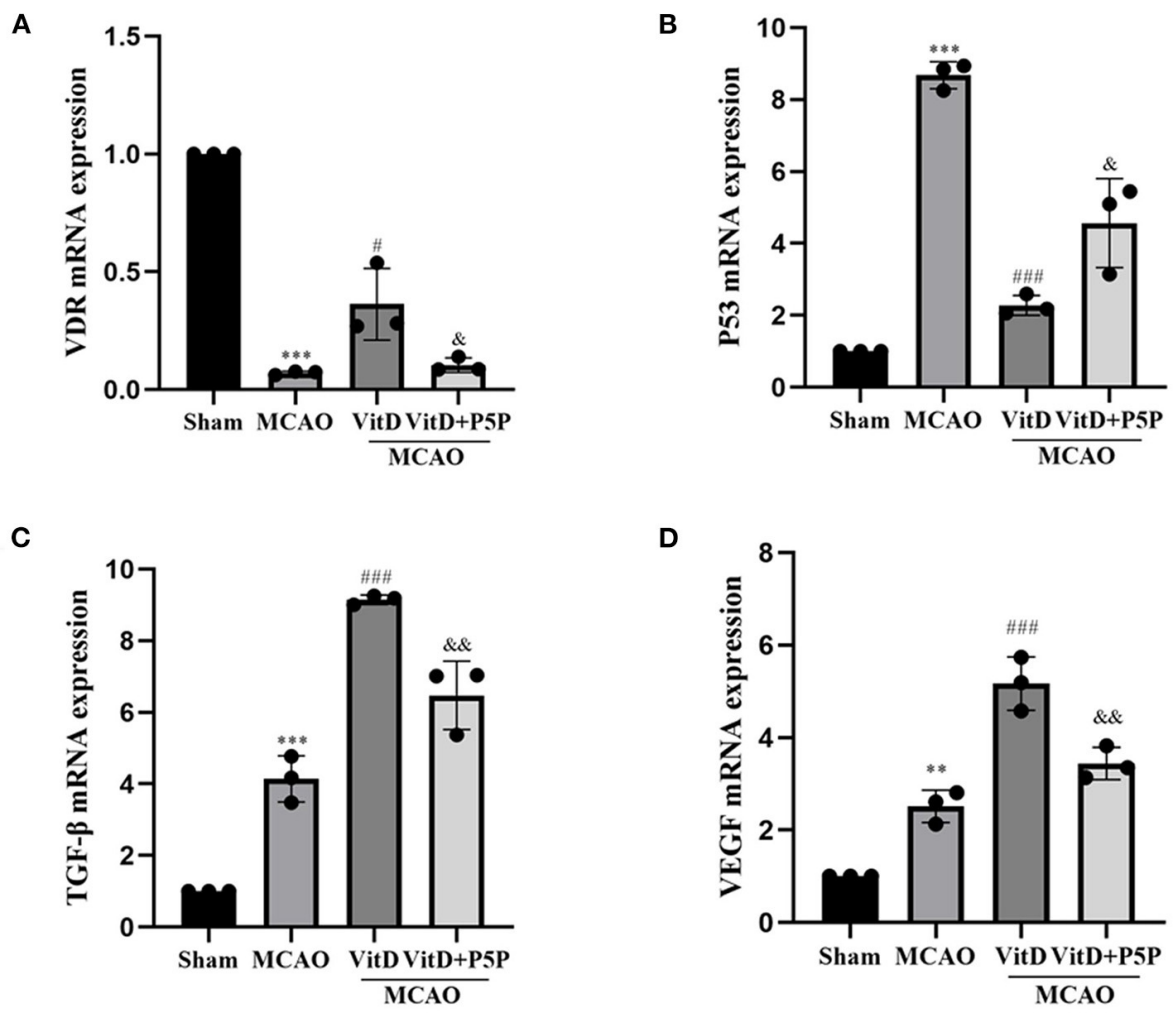
Treatment with 1,25-D3 (Vit D), **(A–D)** the mRNA expression of VDR, VEGF, and TGF-β increased, and the mRNA expression of P53 decreased in the MCAO group. All values are shown as the mean ± SEM. **P < 0.01, ***P < 0.001 (vs. Sham), ^#^*P* < 0.05, ^*###*^*P* < 0.001 (vs. MCAO), ^&^*P* < 0.05, ^&&^*P* < 0.01 (vs. VitD), *n* = 5. P5P, antagonist of VDR; Vit D, (1,25-D3).

The RT-qPCR revealed that the expression levels of P53, VDR, VEGF, and TGF-β mRNA in the peri-infarction cortex after 1,25-D3 treatment were significantly different to those in the MCAO group (P53, F = 78.07, *P* < 0.0001; VDR, F = 92.58, *P* < 0.0001; VEGF, F = 62.85, *P* < 0.0001; TGF-β, F _(3, 8)_ = 106.8, *P* < 0.0001). In the MCAO group, the expression of VDR mRNA was significantly lower (*P* < 0.001), the expression of P53 mRNA was significantly higher (*P* < 0.001), and the expression of VEGF (*P* < 0.01) and TGF-β (*P* < 0.001) mRNA was higher than those in the Sham group. Compared with the MCAO group, 1,25-D3 activated VDR mRNA (*P* < 0.05), inhibited P53 mRNA (*P* < 0.001), and promoted the mRNA expression of TGF-β (*P* < 0.001) and VEGF (*P* < 0.001). P5P partially reversed this process in the 1,25-D3 group (P53, *P* < 0.05; VDR, *P* < 0.05; VEGF, *P* < 0.01; TGF-β, *P* < 0.01; Figure 7). Therefore, it can be concluded that 1,25-D3 promoted VEGF and TGF-β mRNA expression and reduced the expression of apoptotic factor P53 mRNA by activating VDR, thereby promoting angiogenesis after stroke, reducing apoptosis caused by mitochondrial metabolic disorders, and alleviating brain injury.

## Discussion

The present study demonstrated that there is a novel mechanism underlying 1,25-D3 neuroprotection *via* regulation of mitochondrial metabolism to reduce oxidative stress and apoptosis through the AMPK/AKT/GSK-3β signaling pathway in an MCAO model. The main findings are as follows: the administration of 1,25-D3 significantly decreased the neuro-behavior test scores and infarct size after MCAO. The expression of VDR, p-AMPK, p-AKT, and p-GSK-3β were notably upregulated compared with that in the MCAO group at 3 days after reperfusion. The administration of 1,25-D3 significantly reduced the levels of ROS, MDA, and apoptotic factors, including P53, caspase-3, and CytC, but increased the levels of ATP and SDH and promoted the release of TGF-β and VEGF. 1,25-D3 exerted neuroprotective effects by reducing mitochondrial apoptosis and oxidative stress, and promoting angiogenesis induced by cerebral ischemia injury. The inhibition of 1,25-D3 with P5P significantly abolished the angiogenic effects, oxidative stress, and apoptotic effects of 1,25-D3, and worsened the neurological deficits.

First, we assessed the protective effect of 1,25-D3 on cerebral ischemia brain injury using TTC staining and the mNSS score. The mNSS score of the MCAO group was significantly higher than the Sham group, peaked at 24 h after reperfusion, and at 3 days reached the first falling turning point. Treatment with 1,25-D3 significantly decreased the neurological function score compared with the MCAO group. The neurological function score of the P5P group was between the MCAO group and the 1,25-D3 group. TTC staining obviously showed the infarct size of the MCAO group. 1,25-D3 reduced the infarct size, and the infarct size of the P5P group was larger than that of the 1,25-D3 group. Through the pathological sections and staining of brain tissue, we found that the cerebral cortex neurons of the MCAO group showed edema, necrosis of a large number of neurons, and an irregular arrangement of Nissl bodies. After administration of 1,25-D3, neuronal edema was improved, necrosis was reduced, and the number of Nissl substances was increased. These effects were partially reversed by P5P. Both staining and nerve function experiments directly showed that 1,25-D3 improved the nerve function injury. Activation of the AMPK/AKT/GSK-3β signaling pathway has been found to protect the myocardial ischemia-reperfusion injury and enhance glucose homeostasis in the diabetic liver (Yu et al., 2017; Chen et al., 2020). We hypothesized that the AMPK/AKT/GSK-3β signaling pathway might play a similar role in protecting neurons and reducing injury in cerebral ischemia-reperfusion, and focused our research efforts on this aspect.

The AMPK has been reported to be an important energy sensor and regulator of cellular energy utilization. It is activated by oxidative stress and a decrease in cellular energy state (Maslov et al., 2010). The Western blot images indicated that the expression level of activated AMPK in the MCAO group was higher than that in the Sham group and lower than that in the 1,25-D3 group. It has been reported that AMPK activated by some other extracellular stimuli reduces ROS levels by increasing the expression of the antioxidant thioredoxin (Li et al., 2009). The enzyme activities in brain mitochondria were detected using kits, and revealed that the ROS level was decreased after administration of 1,25-D3, which may be caused by AMPK *via* an increase in the antioxidant thioredoxin. Signaling through the phosphatidyl inositol 3-kinase/AKT pathway is known to be protective against the progression of neuronal apoptosis. Phosphatidyl inositol 3-kinase/AKT signaling has been found to play an important role in promoting cell survival, protecting mitochondrial integrity, and inhibiting pro-apoptotic proteins, such as Bax, Bin, and Bad (Hausenloy and Yellon, 2004). Furthermore, AKT signaling is also a vital downstream target of AMPK (Huang et al., 2012; Zhang et al., 2013). AKT plays a key role in some cardiovascular functions, such as angiogenesis (Yu et al., 2012). In this study, compared with the MCAO group, the AKT/GSK-3β signaling pathway was activated and the expression levels of TGF-β and VEGF were increased in the 1,25-D3 group, which may be a result of AKT-induced promotion of angiogenesis. Another effect of AKT is the enhancement of cell survival by inhibition of GSK-3β. GSK-3β, a universally expressed serine/threonine kinase, is considered to be a vital regulator of mitochondrial function and cellular apoptosis during ischemia-reperfusion injury (Gomez et al., 2008; Juhaszova et al., 2009). GSK-3β is a major substrate of AKT, and plays a key role in apoptotic signaling and inflammation (Liu et al., 2010). Previous studies have demonstrated that GSK-3β might be regulated by phosphorylation of its specific serine 9, tyrosine 216, and amino acid residues (Juhaszova et al., 2009). Furthermore, AKT has been shown to be a key upstream signaling factor that phosphorylates GSK-3β at serine 9; this leads to the inactivation of GSK-3β, which further inhibits cardiomyocyte apoptosis and confers cardiac protection (Murphy and Steenbergen, 2005). The present study demonstrated that inhibition of p-AKT increases GSK-3β and that GSK-3β can activate caspase-3, which induces internucleosomal DNA damage, leading to apoptotic cell death (Ueno et al., 2006). In a previous study, The AKT/GSK-3β cascade acted as an intracellular compensatory feedback regulator that inhibited apoptosis and preserved cardiac function in response to myocardial ischemia-reperfusion injury (Yu et al., 2016). Modulation of AKT/GSK-3β signaling has also been deemed as the key underlying mechanism of other cardiac protective agents (Yu et al., 2016; Duan et al., 2017; Min and Wei, 2017; Zhou et al., 2017). We found that these roles were also relevant in MCAO. Western blot demonstrated higher levels of VDR, p-AMPK, p-AKT, and p-GSK-3β in the 1,25-D3 group than in the MCAO group. In the P5P group, VDR, p-AMPK, p-AKT, and p-GSK-3β levels were lower than those in the 1,25-D3 group, which showed that 1,25-D3 did indeed activate the AMPK/AKT/GSK-3β pathway.

Next, we investigated the protein and mRNA levels of apoptosis factors. Western blotting showed that the levels of p-AMPK, p-AKT, and p-GSK-3β were increased when cerebral infarction occurred and that the apoptotic proteins P53 and CytC were also increased, while the levels of VDR were decreased. Compared with the MCAO group, the levels of p-AMPK, p-AKT, and p-GSK-3β were further increased, P53 and CytC were decreased, and the expression level of VDR was increased in the 1,25-D3 group. The inhibitor P5P prevented the protective effect of 1,25-D3. Immunofluorescence staining showed that the protein levels of CytC and cleaved caspase-3 were increased in the neurons of the infarction area, and were decreased in the 1,25-D3 group, compared with the MCAO group. The administration of P5P inhibited the action of 1,25-D3 to reduce the expression of apoptotic proteins. RT-PCR showed that the expression level of P53mRNA was increased in the MCAO group and was decreased in the 1,25-D3 group. The expression level of P53mRNA in the P5P group was slightly higher than that in the 1,25-D3 group. P53 can induce apoptosis by upregulating the pro-apoptotic protein Bax and downregulating the anti-apoptotic proteins Bcl-2 and Bcl-XL. It can also directly accumulate in the plasma membrane and activate the pro-apoptotic protein Bax to trigger cell apoptosis. In addition, it can form complexes with anti-apoptotic proteins Bcl-2 and Bcl-XL to directly induce the increase of mitochondrial outer membrane permeability, leading to the release of CytC, which activates the caspase cascade reaction (Li et al., 2016). Subsequently, the downstream apoptotic protein caspase-3 is successively activated, causing cell apoptosis. Both cerebral ischemia and hypoxia can promote the expression of P53. It has been reported that the release of CytC from mitochondria to the cytoplasm is a common manifestation of various apoptosis modes. A previous study found that nuclear accumulation of GSK-3β and P53 further activated pro-apoptotic genes (Zhou et al., 2019). This evidence was further supported by lithium inhibition of GSK3, which prevented the P53-mediated increase in pro-apoptotic proteins P21 and Bax, as well as CytC release and caspase-3 activation (Watcharasit et al., 2003). Inhibition of GSK3β-P53 signaling provided a survival advantage to renal tubular epithelial cells with renal I/R injury by inhibiting mitochondrial fission (Li et al., 2019).

The above-mentioned studies highlight the role of the GSK-3β/P53 pathway in mitochondrial apoptosis. Other experimental results demonstrated that the role of Bax/CytC/caspase-3 mitochondrial apoptosis pathway mediated by P53 in ischemic stroke rats was observed by establishing a cerebral ischemia-reperfusion injury model and simulating the environment of ischemic stroke neuron injury *in vivo*. We found that the size of cerebral infarction in the model group was larger, with severe cerebral edema, more Nissl-positive cells, decreased NeuN staining, and significantly higher expression of the mitochondrial apoptosis pathway proteins Bax, CytC, and caspase-3. All the above-mentioned studies indicate that the mitochondrial apoptosis pathway is activated after ischemic stroke, which causes neuronal apoptosis in ischemic brain tissue (Xie et al., 2018). In this study, we came to a similar conclusion and found that the expression levels of apoptotic proteins P53, CytC, and caspase-3 were increased after MCAO. However, administration with 1,25-D3 increased the expression of p-GSK-3β, leading to the inactivation of GSK-3β, as well as inhibition of the apoptosis of the GSK3β/P53 signaling pathway, a decrease in the expression of P53, CytC, and caspase-3, a reduction in infarct size and brain edema, and an increase in the number of Nissl-positive cells. The inhibitor P5P partially blocked the anti-apoptotic effect of 1,25-D3. Therefore, we confirmed that 1,25-D3 inhibited the mitochondrial apoptosis pathway, protected neurons, and alleviated brain injury.

After investigating mitochondrial apoptotic proteins, we detected the activities of mitochondrial enzymes to determine the effect of 1,25-D3 on oxidative stress. The results showed that when cerebral infarction occurred, the levels of ROS and MDA in the brain increased, while the levels of ATP and SDH decreased. After the 1,25-D3 treatment, the levels of ROS and MDA decreased, while the levels of ATP and SDH increased. The inhibitor P5P changed the activities of mitochondrial enzymes, levels of ATP and SDH were lower than in the 1,25-D3 group, and ROS and MDA levels were higher than in the 1,25-D3 group. When an ischemic stroke occurs, mitochondria are damaged, energy synthesis is impaired, ATP in brain tissue is rapidly reduced, and the damaged mitochondria release many pro-apoptotic substances, thereby activating the mitochondrial apoptosis pathway and causing the caspase cascade reaction. Eventually, the proteins in cells are degraded, which leads to cell death. A variety of pathological factors lead to abnormal oxidative phosphorylation in mitochondria, such as hypoxia and calcium overload. A sharp increase in ROS, a decrease in scavenging ability, and a large accumulation of ROS in mitochondria induce an oxidative stress response, which further aggravates mitochondrial damage and eventually induces cell death (Hamacher-Brady and Brady, 2016). MDA is the product of mitochondrial membrane lipid peroxidation caused by oxygen free radicals, and its content change corresponds to the process of lipid peroxidation. Therefore, MDA content can be used as an indicator to reflect the level of mitochondrial oxidative stress and to indirectly reflect the degree of mitochondrial damage (Min and Wei, 2017). SDH, as a key enzyme of the tricarboxylic acid cycle, is one of the marker enzymes that reflect mitochondrial function, the activity of which can indirectly reflect the operation degree of the mitochondrial tricarboxylic acid cycle. In the present study, we found that the content of ROS and MDA increased and the activities of ATPase and SDH decreased in the MCAO group, while the levels of ROS and MDA decreased and the activities of ATPase and SDH increased in the 1,25-D3 group. These results show that 1,25-D3 can protect against oxidative stress, reduce energy synthesis disorder, regulate mitochondrial metabolic function, and ultimately protect against brain injury.

Our study has several limitations that should be noted. Only the effect of 1,25-D3 on mitochondrial metabolic function was studied, and the ultrastructure of mitochondria was not observed. We only investigated the effect of 1,25-D3 on the AMPK/AKT/GSK-3β pathway in mediating mitochondrial apoptosis, and other pathways in stroke should be studied in subsequent experiments. Future studies are needed to further explore the potential effects of 1,25-D3 on different periods of stroke, as well as on the recovery phase.

In conclusion, our results revealed that 1,25-D3 can activate VDR to affect angiogenesis, activate the AMPK/AKT/GSK-3β pathway, inhibit apoptotic proteins p53, CytC, and caspase-3, regulate mitochondrial metabolic function, reduce the levels of ROS and MDA, and increase the activity of ATPase and SDH, which ultimately had a protective effect on stroke. As the effects of 1,25-D3 are slowly uncovered, it seems possible that 1,25-D3 is a neuroprotective agent and might be an effective drug for the treatment of ischemic stroke and other diseases in the near future.

## Data availability statement

The raw data supporting the conclusions of this article will be made available by the authors, without undue reservation.

## Ethics statement

The animal study was reviewed and approved by the Ethics Committee for the Use of Experimental Animals at Weifang Medical University.

## Author contributions

YL, XL, and YW were made conception and design of experiments. YL was drafting the article and performed most of the experiments. SX, YZ, MP, XZ, and XW conducted experiments. YW approved the final manuscript. All authors were made manuscript reviews and revisions. All authors contributed to the article and approved the submitted version.

## Funding

This work was supported by the National Natural Science Foundation of China (No. 81870943) and Yuan Du Scholars.

## Conflict of interest

The authors declare that the research was conducted in the absence of any commercial or financial relationships that could be construed as a potential conflict of interest.

## Publisher's note

All claims expressed in this article are solely those of the authors and do not necessarily represent those of their affiliated organizations, or those of the publisher, the editors and the reviewers. Any product that may be evaluated in this article, or claim that may be made by its manufacturer, is not guaranteed or endorsed by the publisher.
